# Special Issue “Nitric Oxide Signaling in Plants”

**DOI:** 10.3390/ijms262010059

**Published:** 2025-10-16

**Authors:** Moon-Sub Lee, Bong-Gyu Mun

**Affiliations:** 1 Department of Crop Science, Chungbuk National University, Cheongju 28644, Republic of Korea; mlee128@chungbuk.ac.kr; 2 Department of Environmental and Biological Chemistry, Chungbuk National University, Cheongju 28644, Republic of Korea

## 1. Introduction

Nitric oxide (NO) has emerged over the past few decades as a critical signaling molecule in both animal and plant systems. NO is now recognized as a vital signaling molecule participating in a wide range of physiological and developmental processes including seed germination, root organogenesis, stomatal behavior, flowering, senescence, and responses to biotic and abiotic stress [1,2]. Despite being a small, simple molecule and a gaseous free radical, NO exerts complex regulatory control by modulating hormonal signaling, redox homeostasis, and ion channel activity and regulating transcriptional and post-transcriptional events such as S-nitrosation, tyrosine nitration, and metal nitrosylation. The first compelling evidence for NO’s role in plant biology appeared in the 1990s. Early studies of NO demonstrated its involvement in pathogen defense, stomatal closure, and stress responses. The field of plant nitric oxide signaling has evolved rapidly, and numerous reports have highlighted how NO interacts with phytohormones including abscisic acid, auxin, and ethylene, as well as with reactive oxygen species (ROS), to form a tightly coordinated signaling network. This intricate crosstalk allows plants to fine-tune growth and development while simultaneously adapting to fluctuating environmental conditions [3,4]. Recent studies continue to uncover new roles for NO in the regulation of stress responses and developmental transitions. For example, NO has been implicated in enhancing tolerance to salinity, drought, hypoxia, and temperature extremes, often by regulating antioxidant defense, osmolyte accumulation, and hormone signaling pathways. Similarly, NO contributes to the release of seed dormancy and the promotion of germination by interacting with gibberellin and abscisic acid signaling. These findings illustrate the versatility of NO as a signaling hub that bridges endogenous developmental programs with environmental stress adaptation [5,6]. Importantly, the study of NO has moved beyond fundamental physiology to reveal significant translational potential. With global agriculture facing unprecedented challenges—climate change, soil salinization, land degradation, and the urgent demand for sustainable crop production—NO is increasingly viewed as a promising candidate for eco-friendly agricultural technologies. Applications under exploration include its use as a seed priming agent, a biostimulant to enhance stress tolerance, and a metabolic enhancer to improve nutrient use efficiency and yield. Experimental evidence supports the capacity of exogenous NO donors to promote seedling vigor, strengthen resistance against pathogens, and improve tolerance to salinity and drought in several crops [7,8]. Such findings provide a scientific rationale for integrating NO-based strategies into sustainable farming systems. This Special Issue titled “Nitric Oxide Signaling in Plants” was conceived to consolidate contemporary insights and innovations across fundamental, physiological, and applied dimensions of NO signaling. It brings together six research and review articles spanning model systems (*Arabidopsis*, *Lilium*), crop species (*soybean*), wild plants (*Sorbus*, *Sporobolus*), and stress paradigms including salinity, water deficit, dormancy, and germination. These studies illustrate how NO orchestrates complex crosstalk between phytohormones, reactive oxygen species (ROS), and other signaling mediators to modulate plant adaptation and resilience. Importantly, this Special Issue also underscores the translational potential of NO in crop improvement. With global agriculture facing escalating challenges from climate change, soil salinization, and increasing demand for sustainable production, NO emerges as a promising candidate for eco-friendly biostimulants, seed priming agents, and metabolic enhancers. From boosting seed production in native grasses to enhancing salt tolerance in soybean, the work compiled here exemplifies both mechanistic depth and real-world relevance. In the sections that follow, we summarize each contribution to this Special Issue and discuss broader implications for the future of nitric oxide research in plant biology and agriculture.

## 2. Summary of Six Contributions

1. *Arabidopsis* Antiporter Genes as Targets of NO Signalling [9]—This study characterizes 50 antiporter genes in Arabidopsis thaliana, revealing multiple subgroups localized to the plasma membrane, chloroplasts, vacuole, etc. qPCR assays under S-nitroso-L-cysteine (CySNO) treatment showed that genes such as CHX2, RCI4, and ER-ANT1 are strongly upregulated under NO donor exposure, with altered responses in gsnor1-3 mutants, indicating the involvement of S-nitrosoglutathione metabolism in NO-mediated gene regulation.

2. Enhancing Soybean Salt Tolerance with GSNO and Silicon [10]—A study of two soybean cultivars under salinity stress showed that combined treatment with the NO donor GSNO and silicon significantly improved plant height, root development, chlorophyll content, ionic homeostasis (Na^+^/K^+^ balancing), antioxidative enzyme activity, and the expression of stress-responsive genes (e.g., GmNHX1, GmSOS2, GmAKT1).

3. Nitric Oxide Pre-Treatment Advances Bulblet Dormancy Release in Lilium [11]—Pretreatment of lily bulblets with an NO donor accelerated dormancy release and flowering by modulating hormone balance, carbohydrate metabolism, and antioxidant systems, thereby reducing required cold storage periods—which has ecological and economic benefits.

4. Response of Hormones, Reactive Oxygen Species, and NO in Sorbus pohuashanensis Embryo Germination [12]—Investigating salt–alkali stress-induced embryo germination promoted by polyethylene glycol (PEG), this work detailed interactions among endogenous hormones (ABA, GA), ROS, and NO signaling during germination in S. pohuashanensis, elucidating how NO integrates with hormonal and oxidative cues to break dormancy.

5. “Exogenous Nitric Oxide Alleviates Water Deficit and Increases the Seed Production of an Endemic Amazonian Canga Grass” [13] (*Sporobolus multiramosus*). Under severe water deficit, exogenous NO improved water status, photosynthesis, biomass, and seed yield. Anatomical changes in stomatal density, root endodermis thickness, and vascular diameter accompanied proteomic shifts linked to stress resilience and reproductive development.

6. “Nitric Oxide Acts as a Key Signaling Molecule in Plant Development under Stressful Conditions” (review) [14]. This comprehensive review details NO biosynthesis via redox pathways, its crosstalk with reactive oxygen species (ROS), melatonin and hydrogen sulfide, and the modulation of phytohormones. It emphasizes NO’s multifunctional roles across seed germination, morphogenesis, stress mitigation, and defense regulation.

## 3. Broader Context and Emerging Themes

NO is produced through both oxidative and reductive enzymatic routes. Among these, nitrate reductase activity represents a major contributor, while mitochondrial nitrite reduction also plays an important role under specific conditions such as hypoxia. Despite decades of effort, however, canonical nitric oxide synthase (NOS) homologs have not been identified in higher plants, leaving the precise enzymatic origin of NO production unresolved. Nevertheless, the unique biophysical properties of NO allow it to diffuse rapidly across membranes, modulate protein activity through S-nitrosation, and influence gene expression. Beyond these direct effects, NO also exerts control over ion channel function and interfaces with second messengers such as calcium (Ca^2+^) and cyclic GMP (cGMP), embedding it within an expansive signaling network. NO also plays a pivotal role in enabling plants to withstand environmental challenges. Exogenous applications of NO donors have repeatedly been shown to alleviate the detrimental effects of drought, salinity, and oxidative stresses. These protective effects are mediated by the regulation of ion homeostasis—particularly the maintenance of optimal Na^+^/K^+^ balance—as well as by the activation of antioxidative defense systems, including superoxide dismutase (SOD), catalase (CAT), and ascorbate peroxidase (APX). NO further safeguards photosynthetic machinery, enhancing both efficiency and resilience, while also modulating transcriptional reprogramming under stress. A striking example comes from recent studies on Solanum multiramosus, an extremophile species, where NO treatments improved stress resilience, highlighting its potential applications in the conservation and ecological restoration of species inhabiting fragile environments. Equally significant is the capacity of NO to integrate with phytohormonal and redox signaling pathways. NO interacts with abscisic acid (ABA) to mediate stomatal closure and water stress responses, while its crosstalk with gibberellins (GA) contributes to the release of seed dormancy and the promotion of germination. In conjunction with ethylene, NO regulates developmental transitions such as senescence and pathogen defense, whereas its synergistic actions with melatonin have been linked to the stabilization of photosynthetic performance and the mitigation of oxidative damage. NO also interacts intimately with reactive oxygen species (ROS), forming a dynamic redox regulatory module that underpins root system architecture, nutrient signaling, and immune responses. Taken together, these insights position nitric oxide as a versatile and indispensable signaling hub. Its elusive biosynthesis, its capacity to buffer plants against environmental stresses, and its intricate hormonal and redox crosstalk all highlight NO’s unique standing within plant biology. More importantly, these features underscore the translational potential of NO, not only in advancing our fundamental understanding of plant physiology but also in providing tangible strategies for crop improvement and ecosystem management.

## 4. Conclusions

The six articles of this Special Issue collectively expand our mechanistic and applied understanding of nitric oxide signaling in plants. From molecular responses in model plants to practical applications in wild grasses and crop-like species, they underscore NO’s transformative potential in agriculture: improving stress resilience, aiding seed systems, and enhancing crop productivity.

Future priorities should include the following:

Field trials of NO-based treatments across multiple crop species and environments.
The integration of NO signaling with breeding and gene editing strategies.
The development of standardized methods for quantifying NO and its downstream markers.
The exploration of NO-mediated thermomemory and cross-stress priming in crops.

These studies lay fertile ground for NO to become a functional component in sustainable crop science, ecological restoration, and agricultural innovation.

## References

[1] Neill S.J., Desikan R., Hancock J.T. (2003). Nitric oxide signalling in plants. New Phytol..

[2] Fancy N.N., Bahlmann A.K., Loake G.J. (2017). Nitric oxide function in plant abiotic stress. Plant Cell Environ..

[3] Domingos P., Prado A.M., Wong A., Gehring C., Feijó J.A. (2015). Nitric oxide: A multitasked signaling gas in plants. Mol. Plant.

[4] Delledonne M. (2005). NO news is good news for plants. Curr. Opin. Plant Biol..

[5] Nabi R.B.S., Tayade R., Hussain A., Kulkarni K.P., Imran Q.M., Mun B.G., Yun B.W. (2019). Nitric oxide regulates plant responses to drought, salinity, and heavy metal stress. Environ. Exp. Bot..

[6] Corpas F.J., Barroso J.B. (2015). Nitric oxide from a “green” perspective. Nitric Oxide.

[7] Sun C., Zhang Y., Liu L., Liu X., Li B., Jin C., Lin X. (2021). Molecular functions of nitric oxide and its potential applications in horticultural crops. Hortic. Res..

[8] Tahjib-Ul-Arif M., Wei J., Jahan M.S., Hasanuzzaman M., Sabuj S., Zulfiqar F., Chen T., Iqbal M., Dastogeer K.M., Sohag A.A.M. (2022). Exogenous nitric oxide promotes salinity tolerance in plants: A meta-analysis. Front. Plant Sci..

[9] Amir R., Qayyum Z., Hussain S., Yun B.-W., Hussain A., Mun B.-G. (2025). Arabidopsis Antiporter Genes as Targets of NO Signalling: Phylogenetic, Structural, and Expression Analysis. Int. J. Mol. Sci..

[10] Msarie M.W., Methela N.J., Islam M.S., An T.H., Das A.K., Lee D.-S., Mun B.-G., Yun B.-W. (2025). Enhancing Soybean Salt Tolerance with GSNO and Silicon: A Comprehensive Physiological, Biochemical, and Genetic Study. Int. J. Mol. Sci..

[11] Yang C., Xu X., Ali M.M., He X., Guo W., Chen F., Fang S. (2025). Nitric Oxide Pre-Treatment Advances Bulblet Dormancy Release by Mediating Metabolic Changes in Lilium. Int. J. Mol. Sci..

[12] Wang X., Shen H., Yang L. (2024). The Response of Hormones, Reactive Oxygen Species and Nitric Oxide in the Polyethylene-Glycol-Promoted, Salt–Alkali-Stress-Induced Embryo Germination of Sorbus pohuashanensis. Int. J. Mol. Sci..

[13] Boanares D., Da-Silva C.J., Costa K.J.A., Serrão Filgueira J.P., Oliveira M.L., Neto L.P., Gastauer M., Valadares R., Medeiros P.S., Ramos S.J. (2023). Exogenous Nitric Oxide Alleviates Water Deficit and Increases the Seed Production of an Endemic Amazonian Canga Grass. Int. J. Mol. Sci..

[14] Khan M., Ali S., Al Azzawi T.N.I., Yun B.-W. (2023). Nitric Oxide Acts as a Key Signaling Molecule in Plant Development Under Stressful Conditions. Int. J. Mol. Sci..

